# Can Immune Suppression and Epigenome Regulation in Placenta Offer Novel Insights into Cancer Immune Evasion and Immunotherapy Resistance?

**DOI:** 10.3390/epigenomes5030016

**Published:** 2021-07-25

**Authors:** Sultana Mehbuba Hossain, Chiemi F. Lynch-Sutherland, Aniruddha Chatterjee, Erin C. Macaulay, Michael R. Eccles

**Affiliations:** 1 Department of Pathology, Dunedin School of Medicine, University of Otago, Dunedin 9054, New Zealand; mehbuba.hossain@postgrad.otago.ac.nz (S.M.H.); lynch781@student.otago.ac.nz (C.F.L.-S.); aniruddha.chatterjee@otago.ac.nz (A.C.); erin.macaulay@otago.ac.nz (E.C.M.); 2 Maurice Wilkins Centre for Molecular Biodiscovery, Level 2, 3A Symonds Street, Auckland 1010, New Zealand

**Keywords:** cancer, placenta, T-cells, PD-1, PD-L1, CTLA-4, immune system, immunotherapy, epigenetics, DNA methylation, transposable elements

## Abstract

Cancer is the second leading cause of mortality and morbidity in the developed world. Cancer progression involves genetic and epigenetic alterations, accompanied by aggressive changes, such as increased immune evasion, onset of metastasis, and drug resistance. Similar to cancer, DNA hypomethylation, immune suppression, and invasive cell behaviours are also observed in the human placenta. Mechanisms that lead to the acquisition of invasive behaviour, immune evasion, and drug and immunotherapy resistance are presently under intense investigations to improve patient outcomes. Here, we review current knowledge regarding the similarities between immune suppression and epigenome regulation, including the expression of repetitive elements (REs), endogenous retroviruses (ERVs) and transposable elements (TEs) in cells of the placenta and in cancer, which are associated with changes in immune regulation and invasiveness. We explore whether immune suppression and epigenome regulation in placenta offers novel insights into immunotherapy resistance in cancer, and we also discuss the implications and the knowledge gaps relevant to these findings, which are rapidly being accrued in these quite disparate research fields. Finally, we discuss potential linkages between TE, ERV and RE activation and expression, regarding mechanisms of immune regulation in placenta and cancer. A greater understanding of the role of immune suppression and associated epigenome regulation in placenta could help to elucidate some comparable mechanisms operating in cancer, and identify potential new therapeutic targets for treating cancer.

## 1. Introduction

Cancer is the second leading cause of global death, with almost 9.6 million deaths from cancer recorded in 2018, equating to approximately one in six deaths [1]. In general, cancer exhibits a range of hallmarks, including escape from immune destruction and, interestingly, many but not all hallmarks recapitulate features of early embryonic development [2]. During tumour growth, tumour cells encounter hypoxic conditions and undergo metabolic and epigenetic reprogramming to survive. As part of the tumour cell survival, the tumour cells also evade the immune system of the host body. Overall, a number of striking similarities are observed between cancer and the placenta. During pregnancy, invasion of the surrounding uterine epithelial tissue by trophoblast cells occurs, which is induced by hypoxic conditions, so as to establish a blood supply for the fetus. There is also rapid proliferation of embryonic and extra-embryonic cells in early embryogenesis, associated with replication stress, and immune suppression to protect the embryo from the maternal immune system [3,4].

Distinctive epigenetic similarities have also been observed between early human development and cancer. Epigenetics is defined as intrinsic cellular mechanisms that influence gene expression without altering the DNA sequence itself [5]. Therefore, epigenetics relies on chemical modifications, and factors that bind to the DNA sequence and thus regulate transcription. Chromatin structure, non-coding RNAs, enhancers, promoters and insulator interactions, along with intracellular and extracellular signalling molecules all play a key role in gene regulation [6]. Comparatively similar alterations to global DNA methylation levels, and histone post-translational modifications, as well as chromatin structure modifications, and genome accessibility, have been discovered in both the placenta and cancer when compared to healthy somatic tissues. These epigenetic modifications are thought to play crucial roles in facilitating immune escape in both cancer and placenta [7,8]. Moreover, epigenetic mechanisms are increasingly becoming recognized as being involved in modulating the response of tumours to immunotherapy drugs, such as immune checkpoint inhibitors (ICI).

Although there are multiple hallmark similarities observed between cancer and the placenta (reviewed in [2,3]), it remains unclear how invasion and immune suppression in cancer and placenta are ultimately regulated. In this regard, there are common regulatory features observed between cancer and placenta [2]. In this article, we review and explore similarities between cancer and the placenta in terms of immune evasion. We also discuss these in the context of innate and acquired resistance to immune checkpoint inhibitors used in cancer therapy.

## 2. Immune Regulation in the Mammalian Placenta and Pregnancy

**Figure 1:**
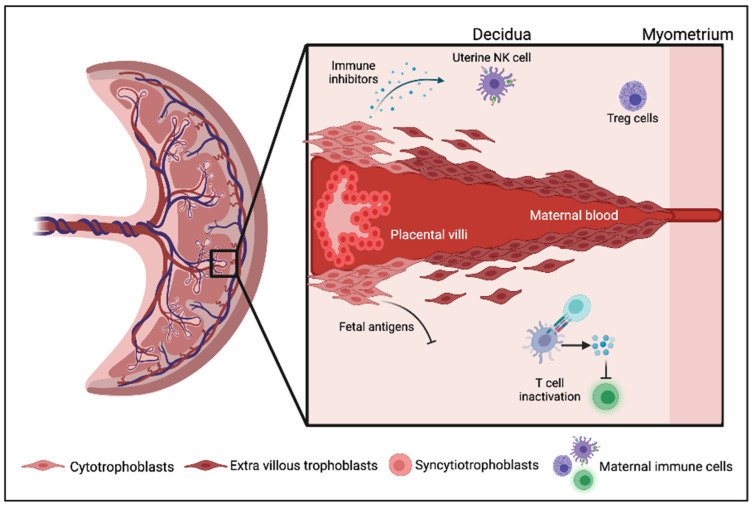
Schematic illustration of the human placenta: The decidua is an important juncture point in the uterine tissue. Inside the decidua, the placental villi, which comprise cytotrophoblast and extravillous trophoblast (EVT) cells, are in close contact with the uterus. The cryptoblast differentiates between the syncytiotrophoblast and the EVT cells. The presence of fetal-derived paternal genetic material can trigger an immune mediated attack against the fetus. However, the trophoblast cells modify endometrial leukocytes to support placental and fetal development.

The main features involving immune regulation in the placenta and pregnancy have previously been reviewed [9,10]. In humans, the placenta and pregnant uterus play a key role in protecting the fetus from the maternal immune system. This is achieved due to the growth factors in the pregnant uterus inducing fetomaternal tolerance, and remodelling of the maternal vasculature to supply oxygen and nutrients to the developing embryo [11]. Prior to formation of the placenta, maternal uterine tissue, known as decidua, plays a vital role in protecting the embryo from maternal immune cells, and it also performs angiogenesis in the decidualizing endometrium to supply adequate nutrition for the development of the embryo. Decidualized cells are formed from the mother’s stromal fibroblast cells which undergo mesenchymal to epithelial transition. Several steps occur in the post-ovulatory or decidualization process, such as secretory transformation of the uterine glands, influx of specialized uterine natural killer (uNK) cells, vascular remodelling and epithelioid transformation of the endometrial stromal cells into highly specialized decidual cells [9] which is critical for the modulation of trophoblast invasion and formation of the placenta (Figure 1). The placenta generates epithelial trophoblast cells in order to proliferate, migrate, and invade the pregnant uterus and its vasculature, in order to supply nutrients through decidual glandular secretions for the development of the fetus [10].

## 3. Mechanisms Underlying Immunosuppression in Cancer and the Placenta

**Figure 2:**
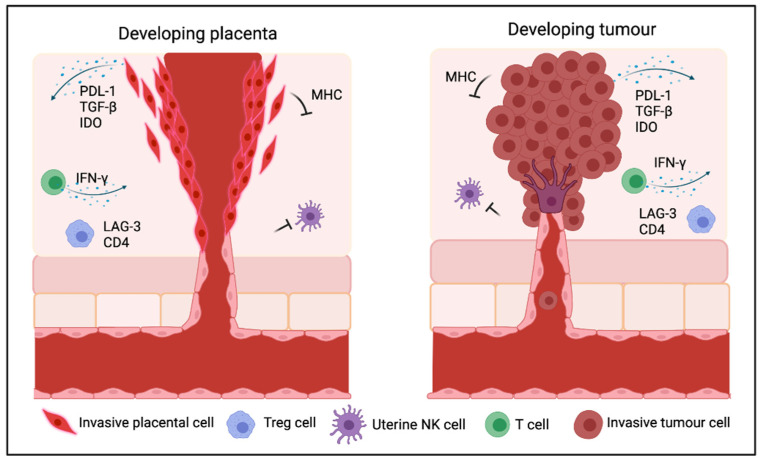
Immune interactions in the developing placenta during pregnancy, and in solid tumours. Several regulatory mechanisms are activated in placenta and solid tumour to create a favourable immunological environment for the development of fetus, or for cancer development. The solid tumour recapitulates several features of early embryos, including the formation of new vessels, the ability to invade surrounding tissues and to evade the immune responses.

Cancer immune evasion, as well as immune suppression in the placenta, involves multiple signalling events, including immunosuppressive cytokines, as well as immune cells, such as regulatory T cells (Tregs), which may be modulated by inherent genetic instability and by immunogenic neoantigen production [12,13]. Immune cells in the placenta play important roles in placental function, where immune suppression is associated with global methylation loss [14]. In contrast to the developing and growing fetus, cancer growth is dependent on controlling immune cells in the tumour microenvironment (TME), and interactions between the malignant cells and the TME [15]. Figure 2 depicts a number of similarities involving immune cell types and molecules between cancer and the placenta.

Initiation of an immune response is determined by the ratio of activated effector T cells (Teffs) and Tregs. Briefly, cell surface antigens (called human leukocyte antigens, or HLAs), are generated by healthy and cancer cells, and are captured by dendritic cells (DCs) and presented on the major histocompatibility complex (MHC, MHCI and MHCII) molecules to antigen-presenting cells (APCs). This causes the priming and activation of Teffs, while the Tregs can regulate an immune response against tumour cells [16,17]. However, cancer cells produce large numbers of neoantigens that trigger the activation of Tregs, and also a subset of immature DCs, promoting the stimulation of Tregs in a transforming growth factor-β (TGF-β)-dependent manner. Cancer cells recruit immature DCs to secrete bioactive TGF-β to stimulate Treg cell proliferation. Additionally, neoantigen production primes activation of Teffs and CD8^+^ T cells. Cancers that possess many CD4^+^ Treg cells, expressing the transcription factor forkhead box P3 (FOXP3), are highly immunosuppressive in nature [18,19], and a high Treg cell to CD8^+^ T cell ratio in the TME is associated with a poor prognosis [20,21]. Tregs suppress Teff cells, promoting tumour malignancy and therapeutic resistance [22], such as in melanoma [21,23,24,25,26,27]. Further, secreted TGF-β plays a role in epithelial–mesenchymal transition (EMT) [28] and contributes to cancer invasion and dissemination. Moreover, TGF-β1 inhibits T cell proliferation, particularly CD8^+^ T-cell proliferation through the canonical SMAD3 signalling pathway. TGF-β also negatively regulates natural killer (NK) cell functions by inhibiting mTOR signalling [29]. Interestingly, placenta also follows a similar pathway of immune escape. Placenta activates immune tolerance towards the fetus by activating macrophages [16,17], decidual (d)NK cells, CD4^+^ and CD8^+^ Tregs, and regulatory B cells [30] and also by expressing TGF-β1 in the human endometrium and placenta [31,32], which is crucial for SMAD transcription factor mediated trophoblast cell invasion [31,33,34].

Cancer progression involves complex interactions between the malignant cells and immune cells in the TME [15]. Tumour cells acquire mechanisms to avoid immune attack by hijacking several epigenetic regulators to modulate gene expression involved in regulating cell identity and controlling cell fate during differentiation [35,36]. The surrounding niche of a cancer, that is, the TME, is composed of fibroblasts, endothelial cells, extracellular matrix (ECM), various immune cells, such as tumour infiltrating lymphocytes (TILs), as well as interactions with human leukocyte antigen class I molecules (HLA-1) expressed on tumour cells, and other tumour-associated factors, such as PD-L1, which together play a crucial role in tumour development [37,38,39,40].

Cancers frequently escape immune attack by promoting defective tumour antigen presentation, as well as secretion of immunosuppressive mediators, to induce tolerance and deviant education of immature DCs. This alters the activated T-cells in both peripheral blood and lymph nodes, and leads to the presence of inhibitory signals expressed by TILs in the TME [17,41].

In an allogeneic pregnancy model, programmed cell death 1 ligand 1 (PD-L1) confers fetomaternal tolerance. In the first trimester, PD-L1 is mainly expressed by syncytiotrophoblast and extravillous trophoblast layers of placenta, while programmed cell death 1 ligand 2 (PD-L2) expression is more restricted to villous cytotrophoblast cells. Human decidual tissues highly express programmed cell death protein 1 (PD-1) in T lymphocytes. Likewise, decidual stromal cells and macrophages constitutively express both PD-L1 and PD-L2, but Th1 cytokines are able to further enhance PD-L1 and PD-L2 surface expression [42]. The exact mechanism and role of PD-L1 upregulation in syncytiotrophoblasts, and in maternal immune suppression remains unclear, although EGFR in syncytiotrophoblasts may participate in regulating PD-L1 expression [43]. PD-L1 is predicted to interact with T cells, cytokines, chemokines and growth factors in the feto-placental environment [44]. Again, trophoblastic cells increase interferon gamma (IFN-γ) expression through microenvironmental stimuli, including CD56+ decidual NK cells, which in turn upregulates PD-L1 expression [45]. Surprisingly, the intermediate trophoblastic cells express low levels of PD-L1 compared to syncytiotrophoblasts [44]. 

Another negative immune checkpoint, cytotoxic T lymphocyte associated protein 4 (CTLA-4), together with indoleamine 2,3-dioxygenase (IDO) act in a co-ordinated fashion to inhibit activated immune responses [46], whereby IDO-mediated local immune suppression protects the fetus *in utero* from the maternal immune system [47]. In the basal decidua, Tregs express CD25, FOXP3 and CTLA-4 [48] and limit allogen-specific Teff cells. Treg cell numbers are elevated in the periphery during early pregnancy [49,50]. IDO1 is overexpressed in many cancers, and in APCs within tumour-draining lymph nodes. In early pregnancy, LAG-3 positive Tregs promote maternal immune tolerance by suppressing Teffs proliferation [51] in the decidua and periphery. In addition, T-cell immunoglobulin mucin-3 (TIM-3) is upregulated by peripheral leukocytes in pregnancy, and is a key element on Th1 cells to reduce proinflammatory Th1-dependent T-cell responses [42,52], but its expression changes in the third trimester of pregnancy. 

Immune evasion in placenta is associated with human chorionic gonadotrophin production by the syncytiotrophoblast layer, followed by down regulation of MHC class I and II antigens, and selective expression of human leukocyte antigen-G (HLA-G) [47]. MHCI molecules bind to killer cell immunoglobulin-like receptors (KIRs), leading to sensitization and activation of NK cells, resulting in NK-mediated cytotoxicity [11]. Through downregulating MHC I expression, the trophoblast cells in both mouse models and in humans lack polymorphic MHC class I and class II antigens, thus dampening NK cell activation. This helps prevent deleterious maternal immune responses against the fetus [11]. HLA-G is a non-classical major histocompatibility complex HLA class I molecule, encompassing >4 membrane-bound (mHLA-G, HLA-G1 to HLA-G4) and 3 soluble (sHLA-G, HLA-G5 to HLA-G7) isoforms [53]. It is involved in maintenance of fetal-maternal immune tolerance, and is mainly expressed throughout all pregnancy trimesters in the invasive extravillous trophoblast cell lineage of the placenta, but not in syncytiotrophoblast or cytotrophoblast cells [44]. HLA-G restricts inhibitory signals by the intermediate trophoblastic cells to control NK cell responses, allowing immunological tolerance of semi-allogeneic fetal tissue [54,55]. HLA-G expression in trophoblasts and PD-L1 expression in syncytiotrophoblasts work together to shield the fetus from immune attack [44]. Furthermore, expression of HLA-G positively correlates with the invasiveness of the trophoblast cells [55]. In cancer, HLA-G expression promotes tumour progression, metastasis and poor clinical outcome. In the TME, HLA-G interacts with receptors expressed on the surface of immune cells, such as immunoglobulin-like transcript 2 (ILT2) on monocytes and B lymphocytes, subsets of DCs, and myeloid derived suppressive cells (MDSCs), NK cells and T cells. HLA-G inhibits NK cells, B cells, and T cells, including CD4^+^ T cells and cytotoxic CD8^+^ T cells [53].

Cancer testis antigens (CTAs), normally expressed in germline stem cells, such as spermatogonia and trophoblasts, are also expressed in cancer [56]. CTA gene promoters (CpG islands) become hypermethylated, leading to tumour immune evasion by abolishing the recognition and response of antigen specific CD8^+^ T cells [56], and also acquired immune resistance in tumour cells [7,57,58]. CTAs play roles in cell differentiation, migration and cell division both in testis and in tumour cells [59]. In melanoma, CTAs are co-expressed with cell surface HLA class I proteins [60,61,62], which may lead to suppression of cytotoxic T lymphocyte (CTLs) and CD8^+^ T cells, as well as suppressing NK cell responses [63].

**Figure 3:**
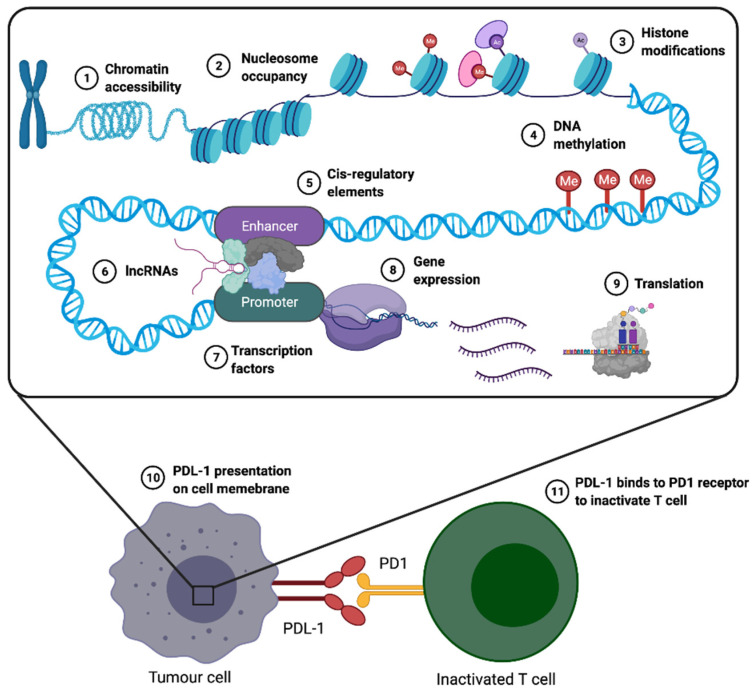
The molecular basis of epigenetic mechanisms and their effect on immune regulation: Genomic DNA is wrapped around histone octamers to form nucleosomes. Histone modification is a covalent post-translational modification that includes methylation, acetylation, phosphorylation, ubiquitination, and many other modifications of the core histones. DNA can be methylated (or hydroxymethylated) at the 5th position on the pyrimidine ring of cytosines in CpG dinucleotides. All of these epigenetic mechanisms play a fundamental role in the dysregulation of T cells, which is associated with binding of PD-1 to PD-L1 to inhibit immune responses, ensuring cancer cell survival and proliferation. The involvement of the PD-1 and PD-L1 interaction is also crucial for immuno-regulatory processes during normal pregnancy.

Epigenetic mechanisms such as chromatin structure modifications and chromatin accessibility, nucleosome occupancy, histone post-translational modifications, DNA methylation, modulation of cis-regulatory elements, long non-coding RNAs (lncRNAs), transcription factors, gene expression and pre- and post-translational regulation (see Figure 3) play a fundamental role in making cancer cells invisible to T cells by dysregulating the antigen-presenting machinery in tumour cells and interfering with T cell activation [7]. 

For instance, DNA methylation levels in gene promoters, and genome-wide methylation levels play important roles in PD-1, PD-L1, PD-L2, and CTLA-4 gene expression, as well as in CD8^+^ T cell exhaustion, tumour-specific immune cell recruitment, impairment of T cell expansion and clonal diversity. As an example, DNMT3 mediates de novo methylation during ICI therapy, while extensive de-methylation of the PD-1 promoter causes permanent CD8^+^ T cell exhaustion [56]. Global methylation loss, mainly occurring in the late-replicating regions occupied by immunomodulatory pathway genes, especially those involved in MHC and cytokine-cytokine receptor interactions, has also been associated with alterations in chromosomal copy number, leading to chromosomal instability, and low anti-tumour immune activity [64]. Chromosomal instability itself influences tumour immune infiltration, and also promotes inflammation by activating the cyclic GMP-AMP synthase-stimulator of interferon genes (cGAS-STING) pathway [65]. Depending on the context, activation of cGAS-STING signalling can paradoxically suppress antitumour immunity and enhance metastasis [66].

## 4. Epigenetic Similarities between Early Human Development and Cancer

DNA methylation and histone modifications are important epigenetic modifications that are involved in embryonic development. They also play a role in tumour development and progression [67,68]. Chromatin structure, non-coding RNAs and the interaction of regulatory elements in *cis* and *trans* work in combination with DNA methylation and histone modifications to regulate transcription. Thus, epigenetic modifications regulate gene expression without changing the original nucleotide sequence of DNA [69].

DNA methylation is one of the most widely studied epigenetic mechanisms. Global DNA methylation patterning contributes to cell identity, and plays a role in the formation of phenotypically distinct cell types that form organs. DNA methylation involves the binding of a methyl group to a cytosine at the C5 position, and occurs predominantly at CpG dinucleotides [70]. CpG dinucleotides occur when a cytosine base is followed directly by a guanine in the 3′-5′ DNA sequence. Many genes contain regions enriched for CpG dinucleotides, known as CpG islands. The promoter regions of genes frequently overlap with CpG islands and, therefore, expression of such genes can be regulated by DNA methylation [71]. Typically, DNA methylation is associated with the silencing of gene expression. In healthy cells, DNA methylation controls gene expression, maintains genome stability, facilitates genomic imprinting, X-chromosome inactivation, chromosome stabilization, and repression of transposable elements (TEs) [72]. DNA methylation at the transcriptional start site (TSS) is strongly associated with silencing of that particular gene, and this functions through blocking transcription initiation [73]. It can also indirectly regulate gene expression through the methylation of regulatory elements such as enhancers, insulators and lncRNAs [74]. Promoter DNA methylation plays an important role in regulation of gene expression and in maintaining cell type [75]. Alterations of DNA methylation in important regulatory regions, such as TSS, or in enhancers in human cancer, may have an impact on cellular phenotypes. Loss of methylation in CpG islands near to the TSS may enhance the expression level of a particular gene, while hypermethylation in the TSS may inhibit expression of specific genes. Nevertheless, methylation in the gene body does not prevent the activation of genes [76]. Approximately 70% of CpG sites within the genome are methylated in healthy adult somatic tissues. However, both the early embryo and placenta and cancers are often recorded as being hypomethylated in comparison to healthy somatic tissues [77,78].

Nucleosomes are the basic unit of chromatin, and are formed by 147 bp DNA wrapped around H2A, H2B, H3 and H4 (two of each) histones. This chromatin structure regulates gene expression in an ATP-dependent manner by removing or assembling nucleosomes along with the DNA, and also by exchanging histone H2A-H2B dimers with dimers of histone variants [79].

### 4.1. Common Epigenetic Mechanisms Frequently Shared between Placenta and Tumours

**Figure 4:**
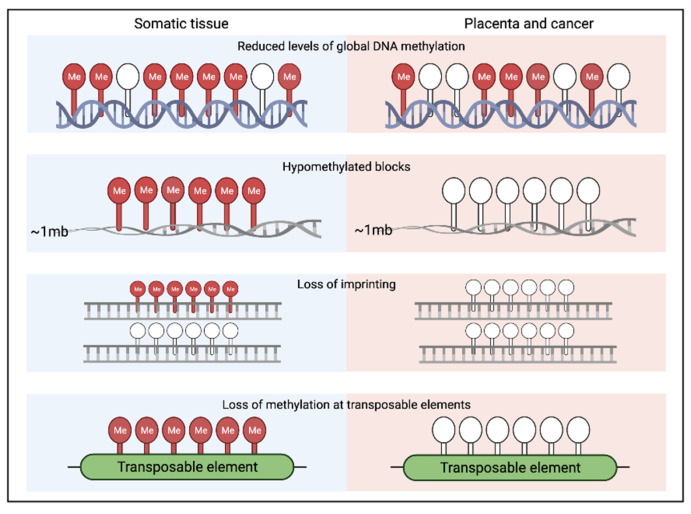
Differences in DNA methylation between somatic tissues, and cancer and the placenta: In healthy tissue, CpG islands in the promoter regions of many genes, including tumour suppressor genes, are unmethylated and active. However, in repetitive regions, such as transposable elements (TEs), in CpG poor intergenic or intragenic regions and in imprinted gene promoter elements, high levels of DNA methylation are usually found, which silence non-coding DNA elements to prevent chromosomal instability. During tumourigenesis, as well as in the placenta, repetitive DNA sequences, TEs and imprinted gene promoters may become hypomethylated, resulting in their aberrant activation.

The placenta demonstrates an approximately 22% reduction in DNA methylation compared to healthy somatic tissue, but it is hypermethylated at known tumour suppressor genes [78,80]. Notably, the placenta shows a DNA methylation landscape more similar to that of tumours than to other healthy somatic tissues [81]. The global CpG methylation level in placenta has been reported at ~58%, compared to somatic tissue types which range from about 70 to 80%, whereas the average CpG methylation level in tumour tissues ranges from about 60 to 65%. Although loss of DNA methylation is a common feature of many cancers [82,83,84], it is important to acknowledge that there are exceptions to this observation. Cancers are extremely heterogeneous and examples exist of tumours that acquire methylation marks, particularly at the promotor regions of tumour suppressor genes. An example of this is the CpG island methylator (CIMP) phenotype observed in some colorectal cancers [85]. Tumour tissues and placenta exhibit a significant level of methylation loss in the majority of repetitive elements, compared to normal somatic tissues [82]. The placenta also exhibits loss of imprinting at some genomic loci, a feature that has also been described in cancer cells [83] (Figure 4). Interestingly, loss of imprinting seems to be a feature that is predominantly observed in humans, a species with an invasive placenta, suggesting that it may play a role in modulating invasion [84]. However, further work would be needed to investigate other species before such conclusions can be drawn. Partially methylated domains are also a feature of both placental and tumour cells however the mechanism that underlies this epigenetic feature remains unknown [86,87].

Comprehensive investigations of the variation in DNA methylation between different tissue types, as well as methylation alterations in tumour tissue, have previously been reported; for example, whole genome bisulfite sequencing (WGBS) was performed on 22 human tissue samples, including healthy tissue, i.e., brain, blood, breast, prostate, liver, lung, colon and placenta specimens and associated tumour tissues [77]. This, and other studies, have observed differences in the global DNA methylation level using matched primary and metastatic samples for melanoma, breast, and colorectal cancer specimens [77,88]. In cancer tissue, 31.1% loss of methylation has been observed within gene promoters, 36.4% loss in intragenic regions, and 32.8% loss located outside of the transcriptional context. The hypomethylated regions have a marked overlap with active regulatory sites. Levels of global DNA methylation progressively decrease during tumourigenesis, such that successive losses of methylation occur from healthy tissue to primary tumours, and then to metastatic tumours. Metastatic tumours exhibited approximately double the level of methylation loss compared to primary tumours [77]. Overall, as tumours progress, DNA methylation levels are frequently diminished to around the same level as that observed in placental tissues.

A comparative study was performed by Nordor et al. (2017) [89] examining DNA methylation similarities between cancer cells and the first trimester placenta. This study identified that on average, 43% of the hypomethylated blocks in placenta overlapped with hypomethylation blocks in five different tumour types, namely bladder urothelial carcinoma, colon adenocarcinoma, head and neck squamous cell carcinoma, pancreatic adenocarcinoma, and rectum adenocarcinoma. This observation is intriguing given that the first trimester of pregnancy is when the placenta is actively invading, and this is when the villous trophoblasts come into contact with the maternal blood. Furthermore, the shared hypomethylated blocks uncovered by Nordor et al. are enriched for genomic regions containing genes that are functionally implicated in several processes, such as immune modulation, EMT and inflammation. Overall, this work demonstrates the utility of using a comparison of the placental and cancer methylomes to narrow down regions that may be functionally relevant in tumourigenesis.

There have been limited comparative studies that aim to assess other epigenetic mechanisms between the placenta and cancer. However, the striking similarities in global DNA methylation patterning highlight the potential of such studies. Some studies have investigated other epigenetic features of genes that have been implicated in both placental and tumour development. This revealed that histone methylation marks (H3K27me3 and H3K9me3) also corresponded with the acquisition of DNA methylation and increased mRNA expression as the pregnancy progressed. This result supports that DNA methylation works in combination with other epigenetic mechanisms to regulate expression in the placenta. Moreover, it supports that the first trimester of placenta may share more features with tumours, and that this may be regulated by shared genes and pathways. Despite this, further epigenetic studies in cancer cells are needed to confirm this result [90]. 

The evidence discussed here provides a strong basis for shared epigenetic features of the placenta and cancer, particularly from a DNA methylation perspective. Furthermore, the numerous shared pathways that have been shown to be activated in both of these tissues, particularly in respect to immune modulation, warrants further investigation. As further technologies such as Chip-Seq, ATAC-Seq and Hi-C become more accessible, it is likely that further epigenetic similarities will be uncovered between these two tissues. 

### 4.2. Epigenetic Features of Embryonic Stem Cells (ESCs), Compared to Tumours and Cancer Stem Cells (CSCs)

DNA methylation is a crucial epigenetic regulator for human embryonic development. The most remarkable genome-wide methylation changes in mammals usually take place in the primordial germ cells and during pre-implantation development. Reduced representation bisulphite sequencing (RRBS) and whole-genome bisulphite sequencing (WGBS) to profile the methylomes of early human embryos from the zygotic stage through to post-implantation [8,91] revealed a downward pattern in DNA methylation from fertilization to the 2-cell stage. The average global level of methylation in the fertilization stage was ~48–54% (sperm 54% and metaphase II oocytes 48%), which decreases to 41% in the zygotes, and further declines to 32% in 2-cell embryos. The lowest level of global methylation (29%) occurs in the blastocyst stage, within the inner cell mass. At this stage, the embryo contains the most pluripotent cells, from which embryonic stem cells (ESCs) are derived. At post-implantation, a sharp gain of methylation level is detected. As embryo development proceeds, genomic regions with a high CpG density, especially nearer to the TSS of genes, tend to be hypomethylated, whereas regions with low CpG density incline towards hypermethylation. 

Cancer stem cells (CSCs) exhibit stem cell-like self-replicating ability, and possess a robust ability to repopulate a tumour mass. They have very similar epigenetic features in common with ESCs, based on biomarkers, gene signatures, signalling pathways, and epigenetic regulators in pluripotency and differentiation potential [92]. For instance, Prominin 1 or CD133 is a CSC biomarker, which is highly expressed in breast cancer, ovarian cancer, colorectal cancer, and glioblastoma as well as in ESCs and human preimplantation embryos [93,94]. The *CD133* gene, both in CSCs and ESCs, uses p53, MAPK/PI3K, and Wnt signalling pathways to control cell proliferation and apoptotic escape [95].

Multiple genes and epigenetic effectors that are epigenetically altered in cancer, have been linked to the maintenance of pluripotency, both in CSCs and ESCs (reviewed in [96]). *SOX9* undergoes demethylation in CSCs to transduce Wnt/β-catenin signals to initiate EMT, which facilitates cancer cell invasion and metastasis [96]. Other genes, such as *OCT4*, *NANOG*, and c-*MYC* in somatic cells, can be reprogrammed through demethylation to achieve stem cell-like properties. ESCs and CSCs both exhibit alterations in *NANOG* expression levels to upregulate *OCT4* expression [97]. However, while ESCs can re-establish homeostasis of their methylation levels in order to transit from pluripotency to a state of differentiation within the three germ cell layers of the embryo [98], CSCs are unable to do this within the TME, although there is evidence that CSCs can be induced to re-establish epigenetic homeostasis and undergo normal fetal development upon being introduced into a blastocyst [99].

The polycomb proteins are epigenetic chromatin modifiers with a crucial role during embryogenesis, which are upregulated and commonly involved in cancer development. These proteins control the silencing of developmental regulators in ESCs and CSCs [100]. Characteristic tumour-specific polycomb marks are frequently associated with methylation and permanent silencing of key regulatory genes in cancer and ESCs through the repressive mark, H3K27me3. These features are suggestive of the presence of a shared regulatory framework, which connects cancer cells with stem/progenitor cell populations [101], and it is hypothesized that epigenetic switching or plasticity may occur in transitioning between these states. Human ESCs have low levels of DNA methylation in the promoter regions of the genes containing H3K27me3 marks, potentially indicating that DNA methylation and H3K27me3 may repress different sets of target genes [8]. 

Another form of epigenetic regulation, that is dependent on nucleosome repositioning by the SWI/SNF remodelling complex, is required for the generation of the pluripotent state for the formation of CSCs. This complex contains ARID1A protein, which can inhibit *SOX2* and *OCT4* gene expression to promote differentiation [102]. This feature is also required for cellular differentiation during embryonic development [103,104].

Therefore, during development of the human embryo, a controlled regulation of pluripotency in reproductive tissues such as the placenta occurs. Chromatin modification signals and global DNA hypomethylation in pluripotent cells, including in ESCs, ensures high plasticity until appropriate differentiation stimuli arrive [105]. However, cancer cells, including CSCs, have lost control over epigenetic regulation of their pluripotent state [10,106].

## 5. Transposable Element (TE) Activation in the Placenta and Cancer; Potential Links to Immune Regulation

TEs comprise more than 45% of the genome, and around 30% of TEs are located in human TSS. The three main classes of TEs are long interspersed nuclear elements (LINEs), short interspersed nuclear elements (SINEs), and endogenous retroviruses (ERVs). These elements have largely arisen from viruses, which have integrated into the genome during evolution. Some TEs have become recruited into tissue-specific genes, and play a role in early human development, but TEs also play a role in disease development [74,106]. TEs are mainly epigenetically silenced in healthy somatic tissues [107], but they become reactivated due to DNA hypomethylation in the placenta during reproduction [108], and in particular disease states, such as in cancer [109]. Active TEs are highly mutagenic, and have the potential to affect the expression of neighbouring genes [110]. 

Functional TEs can change their position within the genome, and can be classified into two groups, depending on the mechanism by which they move to different genomic sites—DNA transposons move directly by a “cut-and-paste” mechanism, while retrotransposons move indirectly through an RNA intermediate (reverse-transcribed into a cDNA copy) [111]. DNA transposons are flanked by terminal inverted repeats, and through the help of a transposase enzyme, the sequence is removed from one region and incorporated into another region in the genome [112]. Retrotransposons are integrated into the genome via an RNA intermediate and unlike DNA transposons, active retroelements retain their original location in the genome, while accumulating copies elsewhere in the genome [113]. Retroelements may be sub-classified based on the presence or absence of long terminal repeats (LTR) in the sequence—i.e., LTR and non-LTR retrotransposons [114]. 

Human endogenous retroviruses (HERVs) represent the LTR group. Full-length HERVs possess LTR regions at each end, which flank a 6–9-kb region of open reading frames (ORFs). The flanking regions encode Gag, Pro, Pol, and Env proteins to facilitate autonomous retrotransposition. Mutational degradation leads to the loss of the ORFs from the structure and almost 90% of HERVs exist as solo LTRs [115].

Non-LTR retrotransposons can be further classified into two subtypes: LINE and SINE retrotransposons. Usually, LINEs are inactive in nature due to 5′ end promoter truncations [114]. The SINEs include Alu and SVA elements. The nonautonomous SINEs utilize LINE-encoded proteins for their retrotransposition, whereas SINEs with SVA elements may also contain LTR sequences [109].

Specific TE subfamilies give rise to TE-derived proteins, new promoters, noncoding RNAs, regulatory elements, and topologically associated domain boundary elements, and are assumed to play a role in transcriptional regulation in a number of biological contexts [116]. Functional TEs are highly active during fertilization of the embryo, where they regulate key pluripotency or totipotency factors by interacting with key developmental genes. TEs interact with pluripotency factors such as NANOG and Oct4 as well as tissue-specific enhancer elements and non-coding RNAs (ncRNAs) to maintain stem cell-like properties in the placenta. TE associated enhancers contribute to early development [117], placentation [108], and innate immune responses [118]. 

The same genes can also play a similar role during oncogenesis. During cancer onset, a number of placental-specific genes undergo reprogramming through the loss of both DNA methylation and repressive chromatin marks, to achieve stem cell-like properties, which resembles similar changes in gene regulation occurring in the placenta [97,109,117]. Moreover, many of the genes that play a role in cell proliferation, invasion, apoptosis, and immunosuppression in cancer are commonly expressed in the placenta [119]. Upon hypomethylation, TEs in cancer become active, and are then capable of targeting protein-coding genes, causing chromosome breakage, and genome rearrangement. TEs are also able to alter splicing and polyadenylation patterns in neighbouring genes, and alter the function of enhancers or promoters [120,121]. In a study by Ye et al. [122], using a mouse model involving CD8^+^ T lymphocytes, the relationship between T lymphocytes and TE expression was compared among different immune cell types. This study revealed that immune cells with the highest enrichment of TE-derived enhancers had an influential role in immune regulatory networks. This study also suggested that epigenetic dysregulation of TE-derived enhancers led to inappropriate activation of immune genes, and these were potentially more prone to reactivate during pathogenesis.

TEs can act as promoters, enhancers, or insulators, and can contribute to the upregulation of specific gene pathways, such as cyclic AMP (cAMP) signalling in the placenta and endometrium [123]. The occurrence of TE-mediated expression of cellular genes is termed onco-exaptation [106]. However, the mechanism by which onco-exaptation occurs still remains unclear. Association of onco-exaptation events with epigenetic reawakening of early developmental TEs, and the reactivation of regulatory TEs in cancer, has recently been extensively reviewed [124]. Nevertheless, how cancer reactivates early developmental pathways through a series of dedifferentiation-associated epigenetic changes, and redirects them to promote malignancy, is presently the subject of intense investigations. Moreover, immune evasion is a very important feature of placentation, and is almost ubiquitous in tumourigenesis [125,126]. There is evidence of a role for TEs in immune regulation; reactivation of TEs stimulates the immune system via viral mimicry and has a positive association with T cell immune infiltration, such as CD8^+^ T cells, in multiple cancer types [121]. However, except for one or two HERV investigations (which are discussed in the following section) relatively little evidence to date links TEs with immune evasion.

## 6. Human Endogenous Retroviruses (HERVs) and Other Repeat Elements; Potential Roles in Immune Modulation

HERVs are a class of TEs, making up approximately 8% of the human genome. HERVs are mainly regulated by epigenetic mechanisms, and the expression of HERVs is negatively correlated with DNA methylation levels in human cells. During early gestation, a high level of HERV expression has been noted, which decreases with increased gestational age, and increased DNA methylation levels [127,128]. Most HERVs are replication incompetent as a result of having sustained numerous mutations and losing relevant genes during evolution [129,130]. Nevertheless, HERVs express high levels of retroviral envelope proteins, supporting the survival of placental cells within an immunosuppressive environment in the presence of maternal immune cells [129]. HERV LTRs are active within the mammalian placenta, as well as the developing embryo, germ cells, and erythroid cells [131].

Highly immunogenic HERVs are easily recognized by endogenous T and B cell responses and are cleared by the immune system. Some HERV epitopes generate an immune response that is too weak to promote antitumor immunity—and these can be categorized into two groups—nonspecific (checkpoint blockade therapy, innate immune agonists) or epitope-specific (vaccination, adoptive T cell therapy) [132]. However, reactivation and expression of HERVs produces antigens that stimulate the immune system by upregulating viral defence mechanism pathways, which has a positive association with the presence of immune infiltrating CD8^+^ T cells [120,121]. In contrast, HERV-encoded *Rec* and *NP9* oncogenes upregulate immunosuppressive β-catenin pathway expression by interacting with promyelocytic leukemia zinc finger protein (PLZF) [133]. During embryonic development, the WNT/β-catenin signalling pathway is also involved in homeostasis, cell migration, haematopoiesis, and wound repair.

In melanoma tumour tissue, activation of WNT/β-catenin signalling in the TME, for example, causes poor T cell infiltration, and is associated with poor innate immunity, especially in the non-T cell inflammatory tumour phenotype, also known as “cold tumours”. Other factors are also associated, such as DCs, interleukin 10 (IL-10), TGF-β, Treg cells, and reduced CD8^+^ T cell priming and infiltration, which together cause immune evasion, and reduced cancer immunosurveillance [134,135]. Furthermore, overexpression of β-catenin inhibits the production of IFN-γ in melanoma cells [136,137].

During exaptation of repetitive elements, intact HERVs substitute open reading frames with 5′ and 3′ LTRs to preserve a residual solo LTR as a promoter or enhancer. In the placenta, these exapted LTRs are involved in the regulation of host IFN pathways. A parallel type of LTR exaptation has also been noticed in cancer cells [118]. Therefore, epigenetic uncontrolled activation of HERVs could have an immune modulatory role in cancer. HERV expression promotes cancer progression through expression of the HERV-encoded *Rec* and *NP9* oncogenes, which along with Env protein, can activate immunosuppressive pathways, or interact with transcription factors. HERV expression causes induction of chromosomal translocations in somatic cells, inactivation of tumour suppressor genes via mutational insertion, homologous recombination, transcription of nearby oncogenes and growth factors via LTRs, all of which can enhance malignancy [130]. Furthermore, reactivation of repeat elements can itself influence epigenetic events, and it remains important to distinguish to what extent this reactivation is a cause, or a consequence of epigenetic changes.

During tumourigenesis, a variety of additional retroelements are reactivated, which then go on to facilitate onco-exaptation, replication stress, retrotransposition, mitotic errors, and deregulation of transcriptional networks, which collectively disrupt genome integrity. All of these repetitive elements are silenced following early human development through DNA hypomethylation, so as to avoid potential genomic instability [138]. Many of these repetitive sequences are also essential for proper mammalian placental development and embryogenesis [138,139], and they interact with host immune cell lineages to adjust immune responses. These repetitive elements also take part in the regulation of developing B and T lymphocytes, through their immunoglobulin and T cell receptor genes for initiating host adaptive immunity [140]. However, the importance of repeat silencing in mammalian tissues remains relatively poorly understood and requires further investigation.

Several studies have found a correlation between activation of repeat elements during tumourigenesis and a role in immunogenicity. Transcripts derived from repetitive elements can stimulate interferon (IFN) and antiviral signalling cascades such as retinoic acid-inducible gene I (RIG-I) receptors and endosomal Toll-like receptors (TLRs), which collectively can establish an antiviral host response or so-called viral mimicry response [120,141,142]. However, activation of an IFN response is cell-type specific [120,141]. Several studies have reported the reactivation of young, replication-competent LINEs in premalignant states, which indicates that early retrotransposon activation could play a role in early cancer onset [143,144].

## 7. Immune Checkpoint Inhibitors (ICIs)

ICIs correspond to a class of drugs that have recently been developed for cancer treatment. These drugs work by leveraging the innate ability of tumour-specific cytotoxic T cells to target and kill cancer cells [145]. While immunotherapy is now used as a first line treatment for cancers such as melanoma, it is also considered as a vital treatment strategy in several types of metastatic cancers such as colorectal, head and neck as well as non-small cell lung cancers (NSCLC) [146]. Although ICIs have revolutionized cancer treatment and substantially improved long-term progression-free survival, the majority of patients exhibit either acquired or innate resistance, which limits the success of treatment. Understanding the myriad causes of resistance, and (re)-sensitizing patients to ICI therapy has become a major area of research in cancer therapy.

In general, ICIs are monoclonal antibodies, designed to disrupt the repression of immune regulatory checkpoints. The main role of the immune checkpoint is to allow tolerance to self. Thus, ICI antibodies bind to immune checkpoint molecules, to block them, which then strengthens the pre-existing antitumour immune responses by activating T–cell responses [147].

Ipilimumab is an ICI antibody, which targets the CTLA-4 signalling pathway. CTLA-4 is expressed intracellularly in Treg cells, and on the cell surface of activated CD8^+^ and CD4^+^ T cells, and Teffs, promoting repression of cell cycle progression of T cells and production of IL-2 and IFN-γ cytokines [148]. CTLA-4 directly competes with CD28 for the ligands CD80 and CD86 and interrupts T cell priming, leading to immunosuppression. CTLA-4 triggers catabolism of the amino acid tryptophan which inhibits T cell activation in cancer [148,149,150]. Additionally, CTLA-4 supresses PI3K/Akt pathways, cyclin D3, cyclin-dependent kinases (cdk4/cdk6) and nuclear transcription factor NF-κB, inhibiting T cell activation [151,152,153]. Hyperactivated/exhausted T cells often cause over-expression of CTLA-4 in intracellular vesicles, inhibiting immune responses against tumour cells [154]. Increased CTLA-4 hyperactivates T cells and inhibits CD28-mediated signalling during antigen presentation [42]. Another mode of action of Ipilimumab is to target a set of regulatory T cells, called Tregs, as has been reported in several clinical trials for metastatic renal cell carcinoma [155]. Tregs have been shown to participate in immunosuppression by downregulating MHC complexes, the shedding of antigens, the induction of immune checkpoints like PD-1 and CTLA-4, reducing co-stimulatory molecules such as GITR and OX40, and the release of various cytokines and factors such as IL-10, VEGF, TGF-β, IDO [41].

Another important immune checkpoint that reduces autoimmunity and promotes self-tolerance is PD-1, which is expressed during T cell immune activation, and which is able to bind to its ligands, PD-L1, and/or PD-L2, present on lymphoid cells, endothelial and epithelial cells, fibroblasts, dendritic cells, and macrophages, exerting immunosuppression [156,157,158]. PD-1 expression on naïve T cells is induced upon T-cell receptor (TCR) activation [159]. PD-L1 is highly expressed in tumour cells and in immune cells, such as DCs, macrophages, MDSCs, and Tregs. PD-L1 interacts with PD-1 and activates the downstream signals of the PD-1 ligand, which in turn causes T cell inactivation (Figure 3). This inactivation inhibits tumour cell killing by the immune system, and allows tumour immune escape [160]. PD-L1 is a membrane bound protein on tumour cells, and other cell types in the TME, whereas PD-1, the corresponding receptor, is expressed on immune cells, such as activated T lymphocytes, B lymphocytes, CD4^+^ and CD8^+^ T-cells, activated monocytes, and dendritic cells [52,161]. PD-L1 expression is regulated by mitogen-activated protein kinase (MAPK) signalling, phosphatidylinositol 3′-kinase (PI3K)/Akt and janus kinase/signal transducers and activators of transcription (JAK/STAT) pathways, hypoxia-inducible factor-1 (HIF1) and NF-κB, as well as epigenetic factors [162]. Elevated expression of PD-L1 on tumour cells is frequently secondary to induction by TILs [163], or driven by driver mutations [164], e.g., mutations in epidermal growth factor receptor (EGFR), via signalling through the PI3K-AKT-STAT3/mTOR pathway, hindering activation of TILs [165]. In melanoma for example, PD-L1 is highly expressed by melanoma cells as an adaptive response to T-cells [13]. Nivolumab and pembrolizumab are categorized as anti-PD1 ICI treatments that bind to PD-1 and block the binding of PD-1 with its ligand PD-L1, in turn causing T cell activation and restoration of antitumour activity [166]. These antibodies tend to be most effective in the presence of a high neoantigen load, because successful anti-tumour immune response requires the reactivation and clonal proliferation of antigen-experienced T cells. The MHC I/II complex variants present tumour-associated peptide antigens on the surface of APCs to reactivate CD8^+^ T cells. TCRs recognize neoantigen–MHC complexes, and signal for T cell activation [167]. However, cancer cells downregulate cell surface expression of MHC classes to facilitate immune escape and avoid T-cell-mediated anti-tumour immunity [168]. To mitigate the effect of downregulated MHC activation of NK cells in tumours, high levels of TGF-β and prostaglandin signalling impair NK-cell function and block their infiltration into the tumour site [169,170]. Additionally, tumour cells’ plasticity allows them to upregulate MHC-I expression temporarily to avoid NK cell recognition, thus facilitating tumour immune escape [171,172]. Moreover, melanoma patients treated with Nivolumab and Pembrolizumab (anti-PD-1) ICI therapy exhibit an increased number of TILs, as well as restoration of the functionality of exhausted T-cells. 

Recently, several alternative immune checkpoints in the tumour microenvironment have been targeted to develop new inhibitors or therapeutic agents (under clinical trial) such as anti-LAG-3 antibody [173] and TIM-3 [174] in an attempt to improve the efficacy of immunotherapy. LAG-3 modulates TCR signalling to prevent excessive lymphocyte activation, and upon antigen stimulation it is expressed on activated CD4^+^ and CD8^+^ T cells [175,176], as well as on a subset of NK cells [177] and on activated Tregs [178]. LAG-3 binds MHC II with higher affinity than CD4, and negatively modulates effector function, and homeostasis of CD8^+^ and CD4^+^ T cells. LAG-3 also mediates T cell exhaustion in combination with PD-1 [179,180]. LAG-3 positive Tregs occur in primary tumours, in lymphocytes of tumour-invaded lymph nodes, and in lymphocytes infiltrating visceral metastases, and produce immunosuppressive cytokines, IL-10 and TGF-β [181]. Galectin-3 and LAG-3 bind together to suppress CD8^+^ T cells, assisting tumour immune evasion [182]. Fibrinogen-like protein 1 (FGL1), elevated in many cancer patients’ plasma, binds with LAG-3 leading to poor prognosis [183]. LAG-3 synergizes with PD-1, often co-expressed in TILs, to inhibit T cells in murine tumour models [184]. LAG-3 and PD-1 expression in CD8^+^ T cells in peripheral blood has been associated with ovarian cancer T cell dysfunction [185]. Another negative immune regulator, TIM-3 contains immunoglobulin and mucin-like domains, and negative regulates CD4^+^ T helper, and CD8^+^ cytotoxic T cells and innate immune cells [186]. TIM-3 regulates Th1 and Th17 by inhibiting expression of proinflammatory cytokines like INF-γ and TNF-α. TIM-3 ligand binds to Galectin-9 (Gal-9) to initiate apoptosis of Th1 and Th17 cells [187]. TIM-3 expression is upregulated in cancer causing T cell exhaustion [188]. TILs (e.g., DCs) bind TIM-3, suppressing innate antitumor immune responses due to tumour-derived nucleic acids [189]. Constitutive expression of TIM-3 in unstimulated peripheral blood CD14^+^ monocytes inhibits both their function, and the activation of CD8^+^ T cells in cancer tissues. TIM-3 expression is induced by TGF-β signalling in tumour associated macrophages (TAM), causing IL-6 secretion [190]. TIM-3 is highly expressed and interacts with CD8^+^ T cells, leading to immune evasion in renal cell carcinoma [191]. PD-1 and TIM-3 are highly expressed by CD8^+^ TILs in advanced melanoma, causing reduced proliferation of T cells and reduced secretion of IFN-γ, IL-2, and TNF-α [192].

Thus, the main objective in using ICI therapy is to block immune checkpoints. These checkpoints in turn regulate inhibitory pathways, favouring the homeostatic balance of the immune system towards maintenance of central/peripheral tolerance and reduction of excessive systemic inflammation in the body [193].

## 8. Immunotherapy Resistance in Cancer, a Common Outcome of ICI Therapy

A proportion of tumours fail to respond to ICI therapy, which is termed innate resistance. In contrast, while initially a number of tumours respond to ICI therapy, after a period of time, a proportion of these tumours progressively develop resistance to ICI treatment and relapse [194]. This is known as acquired resistance, which occurs in a relatively high percentage of patients [195], while linked to this is the inability to robustly predict treatment efficacy and long-term patient response [195,196]. Clinical data show that 20–40% of melanoma patients respond to ICI monotherapies, whereas it has been observed that a higher response rate can frequently be achieved by treating with a combination of ICI therapy drugs simultaneously (there are a number of FDA-approved dual therapies that use two ICI drugs) [197]. The main cause of innate resistance to ICI therapy is the absence of tumour neoantigen presentation, and, therefore, failure to distinguish between tumour cells and normal cells, leading to lack of T cell activation. Additionally, insufficient neoantigen generation and presentation can be due to epigenetic modifications in tumour cells which can change the expression of immune-related genes, such as HERVs, for instance [198]. Treating patients with a combination of checkpoint blockades, e.g., CTLA-4 and PD-1 blockers, may have the effect of increasing a patient’s response to immunotherapy by activating antitumour immune responses in dual pathways. The observed increase in antitumour response rates led to the approval of the Ipilimumab and Nivolumab combination therapy for the treatment of melanoma as well as several other cancers [199]. 

Although ICI therapy is now the standard of care for the treatment of a variety of solid cancers, such as advanced melanoma, head and neck cancer, cervical cancer, renal cell carcinoma, lung cancer, and NSCLC, including tumours with microsatellite instability [200,201], the biggest challenge in ICI therapy is predicting immunotherapy response or resistance in a patient, due to the complex interactions between the immune system and advanced malignancies, such as insufficient generation of T cells, inadequate function of tumour-specific T cells, or impaired formation of memory T-cells. Furthermore, the critical balance between tumour cell-intrinsic factors, tumour cell-extrinsic factors and the heterogeneity in the different tumour lesions in the same patient can modulate the immune response in a patient. Tumour-intrinsic mechanisms of immune evasion include genetic and epigenetic alterations to influence impaired neoantigen formation and presentation, as well as alterations in cellular signalling pathways to disrupt the cytotoxic action of T cells. Tumour-intrinsic factors include the mutation status in *PTEN*, the activity of the WNT/β-catenin signalling pathway, activation of signalling via the cytokine IFN-γ, whether there has been loss of heterozygosity of loci containing genes encoding HLA, and the level of production of neoantigens. Tumour-extrinsic mechanisms involve non-cancerous stromal or immune cells, or other systemic influences (e.g., host microbiota) to promote cancer cell growth and resistance to ICI. The tumour-extrinsic factors include the expression of immune checkpoint molecules, and the formation of desmoplastic tumour stroma to block lymphocyte infiltration [202,203].

Tumour intrinsic and extrinsic factors contribute to primary drug resistance; patients with primary drug resistance to ICI therapy fail to respond to the initial therapy. Tumour intrinsic factors also control the MAPK signalling pathway, leading to production of VEGF and IL-8, which inhibit T cell function. Furthermore, a loss of, or mutation in *PTEN* is correlated with downregulation of IFNγ, granzyme B, and CD8^+^ T cell infiltration [203], while activation of β-catenin signalling pathways can result in T-cell exclusion in melanoma [204]. PTEN deletions, activating PI3K/AKT mutations [205,206], EGFR mutations [207], MYC overexpression [208], and CDK5 disruption [209] result in constitutive PD-L1 expression on cancer cells, leading to the creation of an immunosuppressive environment. Hugo et al. (2017) [210] have identified a set of innate anti-PD-1 resistance signature (IPRES) genes, the expression of which were enriched in tumours of patients exhibiting anti-PD-1 therapy resistance. These genes are involved in the regulation of mesenchymal transition, cell adhesion, ECM remodelling, angiogenesis, and wound-healing.

Following an initial positive response to ICI therapy, acquired resistance is a relatively common outcome in patients with advanced melanoma [211], in spite of initial antitumour T cell activity and recognition of mutational neoantigens [212,213]. ICI therapy can contribute to the creation of a local immunosuppressive environment by activating Tregs, which in turn aggravates T-cell depletion, and causes polarization of immunosuppressive cells and cytokines. As a result, TH1- and TH17-mediated inflammatory responses are stimulated, which leads to activation of oncogenic pathways, and acquisition of resistance to immunotherapy [214]. It has been shown that, after a certain period of treatment, T cells stop exerting cytotoxic effects, even though CD8^+^ T cells are abundantly present during the time of ICI therapy relapse [211]. This might be due to lack of tumour antigen recognition by T cells, the loss of beta-2-microglobulin and HLA (antigen-presenting machinery components), as well as tumour cell–induced or myeloid cell–induced inactivation of T-cell signalling, and activation or a loss of sensitivity to T effector molecules by the cancer cells. Moreover, patients who develop acquired resistance to BRAF inhibitors due to *JAK1* mutations [215] may exhibit cross-resistance to PD-1/PD-L1 inhibitors. This is because *JAK1* mutations modulate the tumour immune microenvironment through the depletion of TILs, which results in IFN-γ insensitivity through epigenetic silencing of interferon-signalling components, or increased expression of negative regulators such as PD-1, TIM-3, CTLA-4, and LAG-3 [211,216,217]. 

## 9. Summary and Future Perspectives

In summary, the placenta, an immune privileged tissue, has similarities to cancer, including in immune evasion pathways and involving genomic as well as epigenomic features, such as global hypomethylation and TE activation. Indeed, global hypomethylation patterns affect a large number of genes in both placenta and cancer, including genes involved in EMT, immune response pathways and inflammation. Although the phenomenon of hypomethylation in cancer cells was described three decades ago, the different mechanisms of hypomethylation that operate in different cell types and in different phenotypes is yet to be fully understood. It is clear that the magnitude of hypomethylation in different cancers can be substantially different. However, the key events that control the level of hypomethylation need to be explored in more detail in future. In addition, although it is now well known that placenta and cancer cells both are hypomethylated, what common functions are achieved in placenta and in cancer via hypomethylation, and how, is yet to be demonstrated. We also described the different layers (e.g., epigenetic and transcriptomic) of regulation of immune cells and the key regulators of immune cells (such as PD-L1). The epigenetic regulation of immune cells has just began to be appreciated. At this stage, our understanding of the epigenetic control of immune cells is broad and somewhat generic. The next decade is likely to see an exploration of epigenetic changes in immune cells within specific contexts (such as in its normal tissue environment versus tumour microenvironments, unique epigenetic changes in different immune cell subsets while interacting with tumour cells, epigenetic changes of certain immune cell subsets during therapy, etc.). If these changes are well demonstrated then the next frontier, and the ultimate goal, will be to find new ways to alter the epigenetic marks for new therapeutic strategies. 

Taken together, the role of epigenetic programming to form the placenta, and epigenetic re-programming that occurs during malignant transformation, and alterations in gene expression [218], could provide mechanisms to allow escape from the host immune system. Thus, a better understanding of the epigenetic mechanisms underlying immunosuppression during pregnancy could offer insights into immune deregulation, and immunotherapy resistance in cancer.
